# A Collaborative Data Collection Scheme Based on Optimal Clustering for Wireless Sensor Networks

**DOI:** 10.3390/s18082487

**Published:** 2018-08-01

**Authors:** Guorui Li, Haobo Chen, Sancheng Peng, Xinguang Li, Cong Wang, Shui Yu, Pengfei Yin

**Affiliations:** 1 School of Computer Science and Engineering, Northeastern University, Shenyang 110819, China; lgr@neuq.edu.cn (G.L.); neu_chenhaobo@163.com (H.C.); congw@neuq.edu.cn (C.W.); 2 Laboratory of Language Engineering and Computing, Guangdong University of Foreign Studies, Guangzhou 510006, China; lxg@gdufs.edu.cn; 3 School of Information Science and Technology, Guangdong University of Foreign Studies, Guangzhou 510006, China; 4 School of Software, University of Technology Sydney, Sydney 2007, Australia; Shui.Yu@uts.edu.au; 5 School of Information Science and Engineering, Central South University, Changsha 410083, China; pppypf@163.com; 6 College of Information Science and Engineering, Jishou University, Jishou 416000, China

**Keywords:** wireless sensor networks, data collection, compressed sensing, clustering, optimization

## Abstract

In recent years, energy-efficient data collection has evolved into the core problem in the resource-constrained Wireless Sensor Networks (WSNs). Different from existing data collection models in WSNs, we propose a collaborative data collection scheme based on optimal clustering to collect the sensed data in an energy-efficient and load-balanced manner. After dividing the data collection process into the intra-cluster data collection step and the inter-cluster data collection step, we model the optimal clustering problem as a separable convex optimization problem and solve it to obtain the analytical solutions of the optimal clustering size and the optimal data transmission radius. Then, we design a Cluster Heads (CHs)-linking algorithm based on the pseudo Hilbert curve to build a CH chain with the goal of collecting the compressed sensed data among CHs in an accumulative way. Furthermore, we also design a distributed cluster-constructing algorithm to construct the clusters around the virtual CHs in a distributed manner. The experimental results show that the proposed method not only reduces the total energy consumption and prolongs the network lifetime, but also effectively balances the distribution of energy consumption among CHs. By comparing it o the existing compression-based and non-compression-based data collection schemes, the average reductions of energy consumption are 17.9% and 67.9%, respectively. Furthermore, the average network lifetime extends no less than 20-times under the same comparison.

## 1. Introduction

Nowadays, wireless sensor networks have emerged as a powerful and low-cost platform for connecting the physical world to the digital world [1]. By deploying numerous tiny and inexpensive sensor nodes in the surveillance area, we can monitor the surrounding environment through periodically collecting the sensed data, which are transmitted back to the sink in a wireless and multi-hop way. Currently, WSNs have found applications in various scenarios, such as environment monitoring, industrial automation, precision agriculture, smart homes, structural health monitoring, military surveillance, and so on [2].

However, the computational power and the energy resources of wireless sensor nodes are strictly constrained. Compared to the data sampling and the data computation, the data communication usually consumes a large portion of energy in wireless sensor nodes. For example, the statistics in [3] demonstrate that the energy consumption of the data communication occupies nearly 86% of the total energy consumption. Therefore, how to reduce the energy consumption of the data communication with constrained computational support is the key problem in wireless sensor networks. Although numerous data collection schemes have been proposed in the literature, the energy efficiency of the periodical data collection and the lifetime of the network still need to be improved [4].

The emerging theory of Compressed Sensing (CS) provides a new paradigm for data collection in WSNs [5]. The compressibility, computational asymmetry, robustness and stability of the compressed sensing theory have made it very suitable for resource-constrained wireless sensor networks. However, it would not be of much benefit to simply apply the compressed sensing theory to data collection in WSNs without considering their features and network structure [6]. Although the tree-based data collection structure can reduce the amount of data transmission, the fault tolerance and the balance of energy consumption are ruined at the same time. Since the cluster-based data collection structure presents many advantages over the flat structure and the tree-based structure [7,8], the CS-based clustered data collection schemes were studied comprehensively in the literature [9,10,11,12,13,14]. By carefully examining these schemes, we identify the following technical challenges that are still posed in the data collection problem of WSNs.

Firstly, how does one determine the optimal cluster size? The number of cluster members has an important influence on the balance of inter-cluster and intra-cluster energy consumption. Whether the cluster head or the cluster member runs out of energy, the data collection in wireless sensor networks is terminated. Secondly, how does one collect the compressed sensed data among cluster heads in an energy-efficient and load-balanced manner? The existing backbone routing tree gives rise to the unbalanced energy consumption in cluster heads. The larger the number of child CHs, the more energy the cluster head consumes. Thirdly, how does one choose the cluster heads and construct the corresponding cluster structure in a distributed manner? In distributed autonomous WSNs, it is usually too costly or even impossible to acquire the specific localizations of all sensor nodes. Thus, only the distributed cluster heads’ election and cluster constructing method is feasible in the real scenario.

Aiming at answering the above questions, we propose a collaborative data collection scheme based on optimal clustering for periodical data collection in WSN. First of all, we divide the data collection process into the intra-cluster data collection step and the inter-cluster data collection step. After evaluating the intra-cluster energy consumption and the inter-cluster energy consumption individually, we model the optimal clustering problem as a separable convex optimization problem and solve it to obtain the analytical solutions of the optimal clustering size and the optimal data transmission radius. Then, we propose a cluster heads-linking algorithm based on the pseudo Hilbert curve to collect the compressed sensed data among cluster heads in an accumulative way. In addition, a distributed cluster-constructing algorithm is also proposed to construct the clusters around virtual cluster heads in a distributed manner. In summary, the main contributions of this paper are listed as follows.

(1) We model the optimal clustering problem as a separable convex optimization problem and solve it analytically to obtain the optimal clustering size and the optimal transmission radius.
(2) We design a cluster heads-linking algorithm based on the pseudo Hilbert curve to collect the compressed sensed data among cluster heads in a collaborative and accumulative manner.
(3) We design a distributed cluster-constructing algorithm to construct the inter-cluster data collection structure around virtual cluster heads in a wireless sensor network.

The remainder of this paper is organized as follows. In Section 2, we summarize the existing CS-based data collection schemes in WSNs. In Section 3, we model the optimal clustering problem and solve it analytically to obtain the optimal network parameters. In Section 4, we present the cluster heads-linking algorithm based on the pseudo Hilbert curve and the distributed cluster-constructing algorithm to construct the underlying data collection structure. In Section 5, we compare and analyse the experiment results of our proposed scheme with other state-of-the-art data collection schemes. Finally, we conclude this paper, as well as discuss our future research in Section 6.

## 2. Related Work

In recent years, the application of compressed sensing theory to data collection in wireless sensor networks has been receiving increasing attention. Bajwa et al. were the first to apply the compressed sensing theory to data collection in wireless sensor networks [15]. They proposed a joint source-channel communication architecture and analysed the relationships among power consumption, distortion and latency for WSNs. However, only single-hop communication was considered, which is not suitable for large-scale wireless sensor networks.

For multi-hop WSNs, Luo et al. proposed the Compressive Data Gathering (CDG) scheme, which was the first complete design to apply the compressed sensing theory to large-scale sensor data collection [16]. After having received the weighted sums of the sensed data from its previous node in the data collection route, each sensor node multiplied its own sensed data with the corresponding coefficients of the measurement matrix and sent the newly-computed weighted sums to its next neighbour node. Other researchers argued that it would not be of much benefit to apply the compressed sensing theory to the data collection problem in WSNs naively [6]. Hence, they proposed a data collection scheme based on hybrid compressed sensing, which treats sensor nodes differently according to their levels in the data collection tree. Furthermore, Xiang et al. formulated the CS-based data collection process as an integer programming problem and solved it with a greedy heuristic algorithm in [17]. Based on the theory of the expander graph and compressed sensing, Zheng et al. constructed a random matrix with a non-uniform distribution and proposed a corresponding random walk routing algorithm to collect the sensed data in WSNs [18].

The characteristics of the cluster structure, such as fault tolerance and traffic loads’ balance, make the CS-based clustered data collection schemes present competitive advantages over other type of data collection schemes. Aiming at minimizing the overall in-network communication and resolving the energy hole problem, Singh et al. designed two data collection schemes based on the compressed sensing theory and the hierarchical clustered structure in [9,10]. In their schemes, sensor nodes carried out different kinds of compression operations according to their levels in the hierarchical structure. In a similar hierarchical data compression scenario, Lan et al. proposed a Compressibility-Based Clustering Algorithm (CBCA), which compresses the sensed data based on the spatial correlation of the readings in the cluster members [11]. In [12], Qiao et al. proposed a compressed data gathering method based on even projection to solve the problem of unstable and unbalanced projection nodes in WSNs with either uniformly- or unevenly-distributed sensor nodes. Zhang et al. substituted the widely-adopted discrete cosine transform basis with the newly-proposed treelet transform basis to sparsify the sensed data and designed the Treelet-based Clustered Compressive Data Aggregation (T-CCDA) scheme in [13]. Furthermore, the morphological watershed transform was also utilized by Hammoudeh et al. in [19] to gather the sensed data in a WSN with a specified level of accuracy in a timely and power-efficient approach.

Furthermore, the spatial and temporal correlation of sensed data can be utilized simultaneously to further improve the data compression ratio of CS-based data collection schemes. In [20], Quan et al. proposed a neighbour-aided compressive data-gathering scheme to collect the spatially- and temporally-correlated sensed data by integrating the structured random matrix with the Kronecker compressed sensing model. Based on the theory of Two-Dimensional Compressive Sensing (2DCS), Wang et al. proposed a new data collection scheme, CS${}^{2}$ collector, to exploit both the spatial and temporal sparsity of the sensed data in WSNs at the same time [3]. Recently, Matrix Completion (MC) and Matrix Approximation (MA), which can be seen as a natural extension to the compressed sensing theory, were also developed to solve the compressive data collection problem in WSNs. By taking advantage of the low-rankness and the short-term stability of the sensed data, Cheng et al. designed the Spatial-Temporal Compressive Data Gathering (STCDG) scheme based on matrix completion in [21]. Based on a similar idea, a correlated spatio-temporal data collection method based on low rank matrix approximation and optimized node sampling was proposed by Piao et al. in [22]. The Gini index was utilized to measure both the spatial distribution of the active nodes and the evenness of the network energy status. In order to obtain the sensed data matrix without missing and corrupted entries, Xie et al. proposed a two-phase data recovery scheme based on matrix completion by exploiting the inherent low-rankness features in the spatio-temporally-correlated sensed data [23].

## 3. System Model and Clustering Analysis

### 3.1. Overview of the Compressed Sensing Theory

Compressed sensing theory (also known as compressive sampling theory or compressive sensing theory) provides a suite of new signal processing principals and techniques for efficiently acquiring and recovering a signal from a set of under-determined linear systems. By effectively exploiting the sparsity of a signal, we can reconstruct it from far fewer samples than required by the classical Shannon–Nyquist sampling theorem [24,25].

According to the compressed sensing theory, the data sensing step and the data compression step can be combined in the following one step:

(1) y=Φx

where $x\in R^{n}$ is the original data, $y\in R^{m}$ is the measurement data and $\Phi \in R^{m\times n}$ is the measurement matrix. Generally, the size of the original data is much larger than that of the measurement data, i.e., n≫m. The usually chosen measurement matrix Φ is a Gaussian or Bernoulli random matrix [26]. It is well known that most natural sensed data are sparse under a certain transform basis [27]. Therefore, the original data *x* can be transformed to *k* sparse data $s\in R^{n}$ as follows:

(2) x=Ψs

where $\Psi \in R^{n\times n}$ is the transform matrix and only *k* elements of *s* have non-zero values. By combining Equations (1) and (2) together, the complete compressed sensing process can be expressed as:

(3) y=As

where $A=\Phi \Psi \in R^{m\times n}$ is referred to as the sensing matrix.

We can recover the original data *x* from the measurement data *y* by solving the following $l_{0}$ minimization problem:

(4) $$\begin{array}{cc} \min_{s} & {∥s∥}_{0}\\ s.t.\; & y=As \end{array}$$

and then transform *s* back to *x* through Equation (2). However, the minimization problem in Equation (4) is NP-hard since all *k*-dimensional subspaces of the *n* dimensions have to be traversed in order to finding the sparsest solution. Usually, we replace the object function in Equation (4) with the convex $l_{1}$ norm of the optimization variable and solve the following $l_{1}$ minimization problem:

(5) $$\begin{array}{cc} \min_{s} & {∥s∥}_{1}\\ s.t.\; & y=As \end{array}$$

It has been proven that the solutions of Equations (4) and (5) are identical with a very high probability as long as the number of measurements *m* satisfies $m\geq cklog\frac{n}{k}$ and the sensing matrix *A* satisfies the following Restricted Isometry Property (RIP):

(6) $$\left(1-\delta_{s}\right){∥s∥}^{2}\leq {∥As∥}^{2}\leq \left(1+\delta_{s}\right){∥s∥}^{2}$$

where *c* is a constant and $\delta_{s}\in \left[0,1\right)$ is the RIP constant [28].

A number of data reconstruction algorithms have been proposed in the literature to solve Equation (5). Essentially, these algorithms can be generally classified into two categories, i.e., the optimization-based algorithms and the greedy pursuit algorithms. In the optimization-based algorithms, different kinds of convex optimization methods are utilized to recover the original data. The representative optimization-based algorithms include the interior-point-based algorithm, the Gradient Projection for Sparse Reconstruction (GPSR) algorithm [29], the homotopy-based algorithm [30], etc. In the greedy pursuit algorithms, different rules of basis selection are designed and applied iteratively to select the optimal support set of the original data. The representative greedy pursuit algorithms include the Orthogonal Matching Pursuit (OMP) algorithm, the Compressive Sampling Matching Pursuit (CoSaMP) algorithm, the Partial Hard Thresholding (PHT) algorithm [31], etc.

### 3.2. System Model

**Figure 1:**
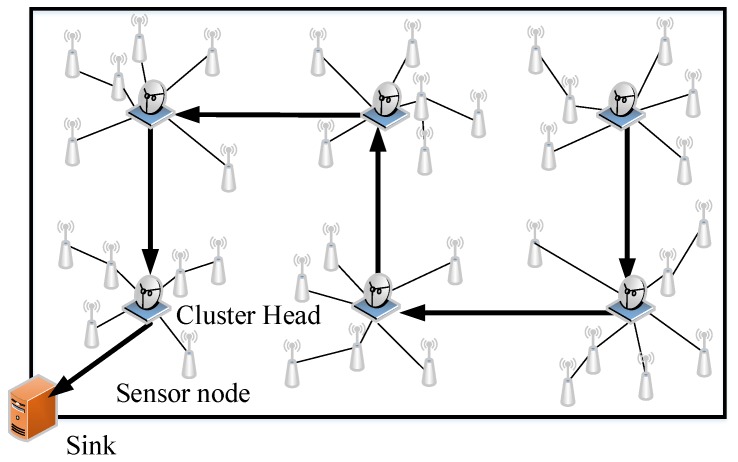
The system model of clustered wireless sensor networks.

The system model of clustered wireless sensor networks is shown in Figure 1. For illustrative purposes, the data transmission process in one round of data collection is divided into the intra-cluster data transmission step and the inter-cluster data transmission step. In the intra-cluster data transmission step, the cluster members send the sensed data to their cluster heads without using the compressed sensing theory. Thus, there is no data compression in the intra-cluster data transmission step. In the inter-cluster data transmission step, each cluster head compresses the sensed data that are received from its cluster members based on the compressed sensing theory. After that, the compressed results are summed with the compressed data of its predecessor in the CH chain and then forwarded to the next cluster head. The above collaborative compression continues until the accumulative compressed results reach the sink.

**Figure 2:**
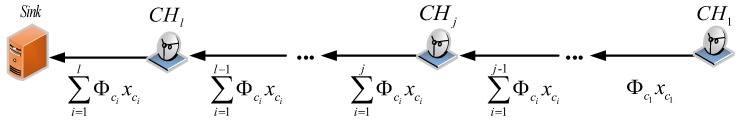
The CS-based data collection process along the CH chain.

In Figure 2, we show the aforementioned inter-cluster data collection step in detail. Assume each cluster head $CH_{j}$ is loaded with the projection vectors $\Phi_{c_{j}}$, which are the corresponding columns of the measurement matrix Φ for its cluster members. Those coefficients can also be generated in situ with a unified seed and the identifiers of its cluster members. After receiving the projection sums of the sensed data $\sum_{i=1}^{j-1}\Phi_{c_{i}}x_{c_{i}}$ from its previous cluster head $CH_{j-1}$, $CH_{j}$ computes the new projection sums $\sum_{i=1}^{j}\Phi_{c_{i}}x_{c_{i}}$ with the projection vectors $\Phi_{c_{j}}$ and the sensed data $x_{c_{j}}$ that are received from its cluster members. The above data compression process starts from the first cluster head $CH_{1}$ and continues until the last cluster head $CH_{l}$ sends the whole projection sums $\sum_{i=1}^{l}\Phi_{c_{i}}x_{c_{i}}$ to the sink, where *l* is the number of cluster heads in the CH chain.

**Figure 3:**
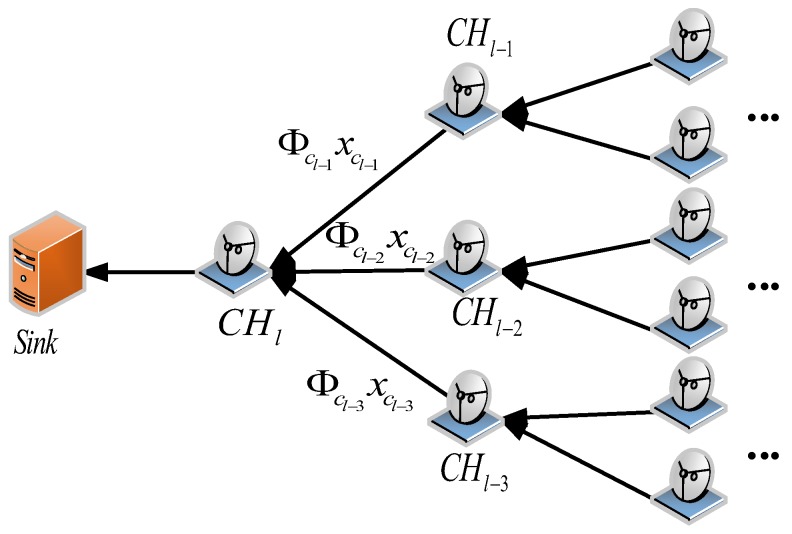
The CS-based data collection process along the CH tree.

It should be noted that the chain-based data collection structure is superior to the tree-based data collection structure in terms of traffic loads’ balance and energy consumption. A tree-based compressed data collection structure is shown in Figure 3. Obviously, the number of received messages of a non-leaf node is in proportion to the number of its child nodes. Thus, the energy consumption of cluster heads with more children is obviously higher than that of less children. Correspondingly, the cluster heads in a tree-based data collection structure usually run out of energy earlier than in a chain-based structure. Therefore, we can avoid the energy hole problem and prolong the network lifetime by collecting the compressed sensed data along the CH chain. Finally, the sink runs the data reconstruction algorithm to recover the original sensed data of the whole network.

### 3.3. Clustering Analysis

We make the following assumptions in the proposed collaborative data collection scheme.

(1) All sensor nodes are randomly distributed in the surveillance area with an independent and identical distribution, which can be modelled as a Poisson point process with parameter λ.
(2) All sensor nodes are set to the same level of data transmission power and data transmission rate. Therefore, the data transmission range of all sensor nodes is identical.
(3) Every sensor node is aware of its location. A number of sensor localization algorithms for WSNs can be used for this purpose [32].

Different from previous research [33], the cluster sizes are identical instead of increasing gradually from the sink towards the network periphery. The CS-based data collection process along the CH chain can balance the energy consumption of all cluster heads and avoid the energy hole problem. Moreover, it is also easy to implement and analyse.

To evaluate the total energy consumption in one round of data collection, we utilize the following energy consumption model, which was also adopted by other researchers [34,35]:

(7) $$E_{t}\left(b,d\right)=\left\{\begin{array}{cc} b(E_{elec}+d^{2}E_{amp})\;, & if\;d\leq d_{0}\\ b(E_{elec}+d^{4}E_{amp})\;, & if\;d>d_{0} \end{array}\right.$$

(8) $$E_{r}\left(b\right)=bE_{elec}$$

In Equation (7), $E_{t}\left(b,d\right)$ denotes the energy consumption for transmitting *b* bits of data to a receiver of *d* meters away. $E_{elec}$ and $E_{amp}$ denote the energy consumption of the transmission circuit and the power amplifying circuit to process one bit of data, respectively. According to the relationship between the distance *d* and the threshold $d_{0}$, the propagation loss can be modelled as either the free-space power loss model or the multiple path fading power loss model. In Equation (8), $E_{r}\left(b\right)$ denotes the energy consumption for receiving *b* bits of data from the transmitter.

**Figure 4:**
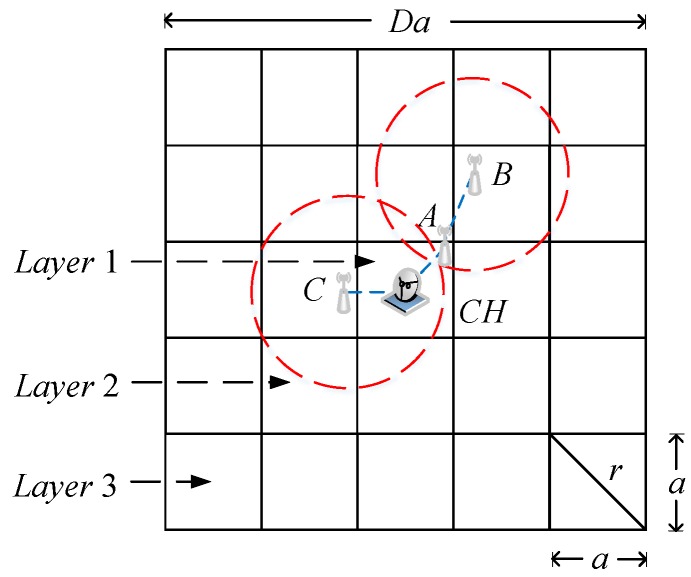
The clustering analysis model.

We show the clustering analysis model in Figure 4, where the whole surveillance area is partitioned into a number of small grids of size a×a. The transmission radius of each sensor node should satisfy $r\geq \sqrt{2}a$. Thus, the sensor nodes that are deployed in the same grid are able to communicate with each other. We stack D×D grids into a square to form a cluster. In order to reduce the total intra-cluster energy consumption, the cluster head should be placed at the centre of the cluster. After compressing the sensed data in the current cluster, cluster heads forward the accumulative compressed projections to the sink along the CH chain.

Suppose there are *n* sensor nodes deployed in the surveillance area and the sensed data of each sensor node can be encoded into a data package of *p* bits. By evaluating the intra-cluster energy consumption and the inter-cluster energy consumption individually, we can formulate the optimal clustering strategy.

(1) The intra-cluster energy consumption:

Considering the sensor node that is in the same grid as the cluster head, only one hop is required to transmit the sensed data to the cluster head. Thus, the transmission energy consumption for one node that is in the centre grid of the cluster (also known as Layer 1) is $p\left(E_{elec}+\delta \left(r\leq r_{d}\right)r^{2}E_{amp}+\delta \left(r>r_{d}\right)r^{4}E_{amp}\right)$, where δ() is the indicator function. For presentation purposes, we only describe the case where the transmission distance *d* is less than or equal to the threshold $d_{0}$. The following analysis can be easily modified to suit the opposite case. Then, the above transmission energy consumption can be simplified to $p\left(E_{elec}+r^{2}E_{amp}\right)$. There are $\lambda a^{2}$ nodes deployed in each grid, where λ is the density of the Poisson point distribution. Thus, the total transmission energy consumption for nodes in the first layer of one cluster is:

(9) $$E_{t-I}^{L1}=p\lambda a^{2}\left(E_{elec}+r^{2}E_{amp}\right)$$

Based on a similar analysis, the total receiving energy consumption for nodes in the first layer of one cluster is:

(10) $$E_{r-I}^{L1}=p\lambda a^{2}E_{elec}$$

Next, we consider the nodes that are in the *h*-th layer of one cluster, where h≥2. There are (D+1)/2 layers in each cluster and 8(h−1) grids in the *h*-th layer. Meanwhile, the number of hops required to transmit the sensed data collected in the *h*-th layer to the cluster head is bounded by ;[h−1,h]. For example, Node B in Layer 2 requires two hops to reach the cluster head CH in Figure 4. However, only one hop is required for Node C to reach CH because Node C is closer to CH than Node B, although they are in the same layer. Therefore, the lower bound and the upper bound of the total transmission energy consumption for nodes in one cluster except the first layer can be evaluated as:

(11) $$\begin{array}{cc} {\underline{E}}_{t-I}^{L2+}= & \sum_{h=2}^{\frac{D+1}{2}}8{\left(h-1\right)}^{2}p\lambda a^{2}\left(E_{elec}+r^{2}E_{amp}\right)\\ = & \frac{D\left(D^{2}-1\right)}{3}p\lambda a^{2}\left(E_{elec}+r^{2}E_{amp}\right) \end{array}$$

and:

(12) $$\begin{array}{cc} {\bar{E}}_{t-I}^{L2+}= & \sum_{h=2}^{\frac{D+1}{2}}8\left(h-1\right)hp\lambda a^{2}\left(E_{elec}+r^{2}E_{amp}\right)\\ = & \frac{\left(D+3\right)\left(D^{2}-1\right)}{3}p\lambda a^{2}\left(E_{elec}+r^{2}E_{amp}\right) \end{array}$$

respectively. Similar to the above analysis, the lower bound and the upper bound of the total receiving energy consumption for nodes in the same area can be evaluated as:

(13) $$\begin{array}{cc} {\underline{E}}_{r-I}^{L2+}= & \sum_{h=2}^{\frac{D+1}{2}}8{\left(h-1\right)}^{2}p\lambda a^{2}E_{elec}\\ = & \frac{D\left(D^{2}-1\right)}{3}p\lambda a^{2}E_{elec} \end{array}$$

and:

(14) $$\begin{array}{cc} {\bar{E}}_{r-I}^{L2+}= & \sum_{h=2}^{\frac{D+1}{2}}8\left(h-1\right)hp\lambda a^{2}E_{elec}\\ = & \frac{\left(D+3\right)\left(D^{2}-1\right)}{3}p\lambda a^{2}E_{elec} \end{array}$$

respectively. For the sake of convenience, we use the lower bounds ${\underline{E}}_{t-I}^{L2+}$ and ${\underline{E}}_{r-I}^{L2+}$ in the following evaluations.

The number of clusters in the surveillance area can be computed as $n/\lambda D^{2}a^{2}$, because there are $\lambda D^{2}a^{2}$ sensor nodes in each cluster. Therefore, the total intra-cluster energy consumption is:

(15) $$\begin{array}{cc} E_{I}= & \left(E_{t-I}^{L1}+E_{r-I}^{L1}+{\underline{E}}_{t-I}^{L2+}+{\underline{E}}_{r-I}^{L2+}\right)\frac{n}{\lambda D^{2}a^{2}}\\ = & \left(\frac{D}{3}-\frac{1}{3D}+\frac{1}{D^{2}}\right)pn\left(2E_{elec}+r^{2}E_{amp}\right)\\ \approx & \left(\frac{D}{3}-\frac{1}{3D}\right)pn\left(2E_{elec}+r^{2}E_{amp}\right) \end{array}$$

(2) The inter-cluster energy consumption:

As shown in the system model, each cluster head compresses the sensed data into a data package of mp bits with the measurement matrix Φ. Then, it sends the accumulative compressed projections to the sink along the CH chain. There are *D* hops between two adjacent cluster heads and $\sqrt{2}D/2$ hops between the sink and the last forwarding cluster head. Thus, the total energy consumption of the inter-cluster data transmission can be evaluated as:

(16) $$E_{t-II}=\left(\frac{n}{\lambda D^{2}a^{2}}-1\right)Dmp\left(E_{elec}+r^{2}E_{amp}\right)+\frac{\sqrt{2}D}{2}mp\left(E_{elec}+r^{2}E_{amp}\right)$$

Similarly, the total energy consumption of the inter-cluster data receiving can be evaluated as:

(17) $$E_{r-II}=\left(\frac{n}{\lambda D^{2}a^{2}}-1\right)DmpE_{elec}+\frac{\sqrt{2}D}{2}mpE_{elec}$$

Therefore, the total inter-cluster energy consumption is:

(18) $$\begin{array}{cc} E_{II}= & E_{t-II}+E_{r-II}\\ = & \left(\frac{n}{\lambda D^{2}a^{2}}-1\right)Dmp\left(2E_{elec}+r^{2}E_{amp}\right)+\frac{\sqrt{2}D}{2}mp\left(2E_{elec}+r^{2}E_{amp}\right) \end{array}$$

(3) The optimal clustering strategy:

By adding the intra-cluster energy consumption Equation (15) and the inter-cluster energy consumption Equation (18) together, we can obtain the total energy consumption in one round of data collection as follows:

(19) $$\begin{array}{cc} E= & E_{I}+E_{II}\\ = & \left(\frac{Dn}{3}-\frac{n}{3D}+\frac{nm}{\lambda Da^{2}}-Dm\right)p\left(2E_{elec}+r^{2}E_{amp}\right)+\frac{\sqrt{2}D}{2}mp\left(2E_{elec}+r^{2}E_{amp}\right)\\ = & \left(\left(\frac{n}{3}-\frac{(2-\sqrt{2})m}{2}\right)D+\left(\frac{nm}{\lambda a^{2}}-\frac{n}{3}\right)\frac{1}{D}\right)p\left(2E_{elec}+r^{2}E_{amp}\right)\\ = & \left(c_{1}D+\frac{c_{2}}{D}\right)c_{3}\left(1+c_{4}r^{2}\right) \end{array}$$

where $c_{1}=n/3-\left(2-\sqrt{2}\right)m/2$, $c_{2}=nm/\lambda a^{2}-n/3$, $c_{3}=2pE_{elec}$ and $c_{4}=E_{amp}/2E_{elec}$.

In order to keep all sensor nodes connected to each other, we should guarantee that at least one additional sensor node is located in the coverage area of each sensor node. Thus, the transmission radius should satisfy $r\geq \sqrt{2/\lambda \pi }$. Furthermore, the maximal value of *D* is obtained when all sensor nodes are in the same cluster, i.e., $D\leq \sqrt{2n/\lambda r^{2}}$. Therefore, the optimal clustering problem can be cast as the following optimization problem:

(20) $$\begin{array}{cc} \min_{D,r} & \left(c_{1}D+\frac{c_{2}}{D}\right)c_{3}\left(1+c_{4}r^{2}\right)\\ s.t.\; & 0<D\leq \sqrt{\frac{2n}{\lambda r^{2}}}\\ & r\geq \sqrt{\frac{2}{\lambda \pi }} \end{array}$$

According to the compressed sensing theory, the size of the original data is much larger than that of the measurement data, i.e., n≫m. Thus, $c_{1}>0$. Meanwhile, the number of compressed sensed data in a cluster is much larger than that of uncompressed sensed data in a small grid. Thus, $c_{2}>0$. It is obvious that $c_{3}>0$ and $c_{4}>0$. Let $f\left(D,r\right)=\left(c_{1}D+c_{2}/D\right)c_{3}\left(1+c_{4}r^{2}\right)$. Since dom(f) is convex and $\nabla^{2}f\left(D,r\right)⪰0$, the optimization problem in Equation (20) is convex. Meanwhile, it is trivial to see that problem in Equation (20) is separable and can be solved by splitting it into the following two independent optimization sub-problems:

(21) $$\begin{array}{cc} \min_{D} & \;c_{1}D+\frac{c_{2}}{D}\\ s.t.\; & 0<D\leq \sqrt{\frac{2n}{\lambda r^{2}}} \end{array}$$

and:

(22) $$\begin{array}{cc} \min_{r} & \;1+c_{4}r^{2}\\ s.t.\; & r\geq \sqrt{\frac{2}{\lambda \pi }} \end{array}$$

The optimal solution of Equation (21) can be obtained by computing the stationary point of the object function and comparing it with the constraint criterion. Thus, the optimal clustering size can be determined as:

(23) $$D^{*}=\left\{\begin{array}{cc} \sqrt{\frac{c_{2}}{c_{1}}} & \left(6-\frac{3\sqrt{2}}{2}\right)m-n<\lambda a^{2}\\ \sqrt{\frac{2n}{\lambda r^{2}}} & otherwise \end{array}\right.$$

It is trivial to obtain the optimal transmission radius $r^{*}=\sqrt{2/\lambda \pi }$ because the objection function of Equation (22) is monotonically increasing.

## 4. The Collaborative Data Collection Scheme

In wireless sensor networks, the sink is aware of the surveillance area in which all sensor nodes are deployed. However, it does not need, nor is it even possible to know, the specific deployment localizations of all sensor nodes. Assume the surveillance area is a rectangle with length *L* and width *W*. After determining the optimal clustering size $D^{*}$ according to Equation (23), the sink generates $l=2LW/{\left(D^{*}r^{*}\right)}^{2}$ virtual cluster heads and sets them to the centre of each cluster individually. Note that these virtual cluster heads are only virtual agents used to build the CH chain, instead of real sensor nodes.

Firstly, the sink runs the cluster heads-linking algorithm based on the pseudo Hilbert curve to build the CH chain. Afterwards, the distributed cluster-constructing algorithm is executed with the goal of selecting the proper cluster heads and forming the underlying data collection structure. Finally, all sensor nodes send the sensed data to the sink by following the same principles that have been explained in detail in the system model. It should be noted that the above two algorithms are only required to be executed once. Therefore, the cost of constructing the underlying data collection structure is trivial in comparison to that of the periodical data collection.

### 4.1. The Cluster Heads-Linking Algorithm Based on the Pseudo Hilbert Curve

By designing a chain-based data collection structure among CHs, the energy consumption of all cluster heads can be well balanced. Essentially, the CH chain is a Hamilton path in the CH graph. It traverses all cluster heads and accesses each cluster head only once. The Hilbert curve is the most popular space-filling curve, which presents the strong locality preserving property and can be used as a Hamilton path for any region of size $2^{q}\times 2^{q}$, where q∈N. However, the surveillance areas of WSNs are usually rectangles of arbitrary sizes instead of squares of exponential sizes. Therefore, we generate a pseudo Hilbert curve of arbitrary dimension for general WSN applications.

**Figure 5:**
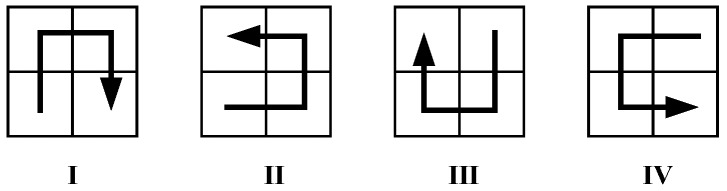
The four primitive Hilbert curves.

A Hilbert curve can be decomposed into a set of primitive curves. In order to connect the end points of all primitive curves correctly, four primitive curves with different orientations are defined and shown in Figure 5. Meanwhile, an orientation transformation rule for different orientations is also defined in Table 1. After partitioning the parent Hilbert curve into four parts P1, P2, P3 and P4 in the order of lower-left, lower-right, upper-left and upper-right, the orientations of each parts $O_{1}$, $O_{2}$, $O_{3}$ and $O_{4}$ can be determined by looking up in the table according to the parent orientation of the curve.

**Figure 6:**
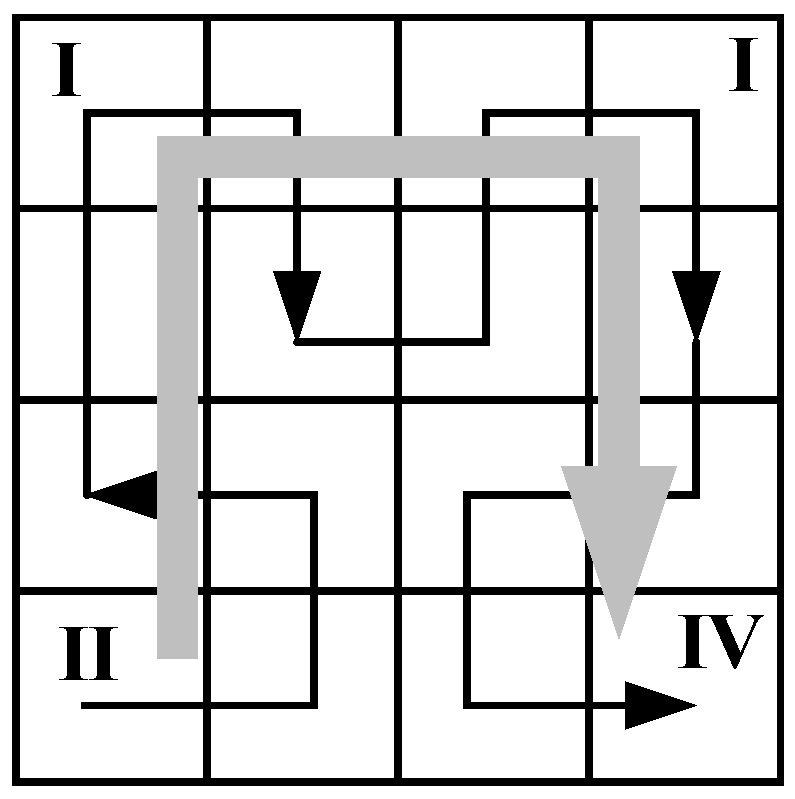
A 4×4 Hilbert curve.

A simple 4×4 Hilbert curve is shown in Figure 6 to demonstrate the aforementioned orientation transformation rule, where the parent orientation is drawn in a bold arrow. Since the parent orientation of the Hilbert curve is I, the lower-left, lower-right, upper-left and upper-right parts of the Hilbert curve are arranged as the primitive Hilbert curves II, IV, I and I, respectively. After connecting the end points of these four parts according to the orientation of the Hilbert curve I, a 4×4 Hilbert curve can be built.

**Figure 7:**
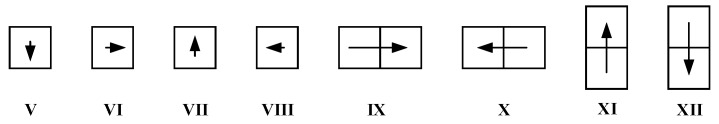
The extra eight primitive pseudo Hilbert curves.

We adopt the primitive Hilbert areas and the primitive Hilbert curves designed by Wu et al. in [36] to build the CH chain. In addition to the original primitive Hilbert curves I to IV, extra eight primitive pseudo Hilbert curves are designed and shown in Figure 7. Since the horizontal and vertical lengths of all primitive Hilbert curves are at most two units, we first partition the whole surveillance area into a number of primitive Hilbert areas, the length of each side of which is less than three units. Then, we fill each sub-area with a primitive (pseudo) Hilbert curve based on its size, orientation and the orientation transformation rule. In order to build the whole space-filling curve, we connect all primitive (pseudo) Hilbert curves head-to-tail in sequence. Finally, we traverse the resultant pseudo Hilbert curve to record the indexes of all cluster heads with the goal of forwarding the compressed data along the CH chain in the inter-cluster data transmission step. The pseudo code of the cluster heads-linking algorithm based on the pseudo Hilbert curve is shown in Algorithm 1. Please refer to [36] for the pseudo Hilbert curve generation details.

**Algorithm 1** Cluster heads-linking algorithm based on the pseudo Hilbert curve.
**Input:** length *L*, width *W*, the number of CHs *l***Output:** locations and indexes of virtual CHs1:Partitions the surveillance area into a set of squared sub-areas2:**while** the length of each side of the primitive area ≥ 3 **do**3: Partitions each squared sub-area into a set of primitive Hilbert areas4:
**end while**
5:**for all**i∈ {primitive areas} **do**6: Fills *i* with a primitive (pseudo) Hilbert curve I to XII7:
**end for**
8:**for all**j∈ {primitive (pseudo) Hilbert curves} **do**9: Connects the starting point of *j* with the end point of its predecessor10:
**end for**
11:Traverses the pseudo Hilbert curve and records the indexes of all cluster heads


**Figure 8:**
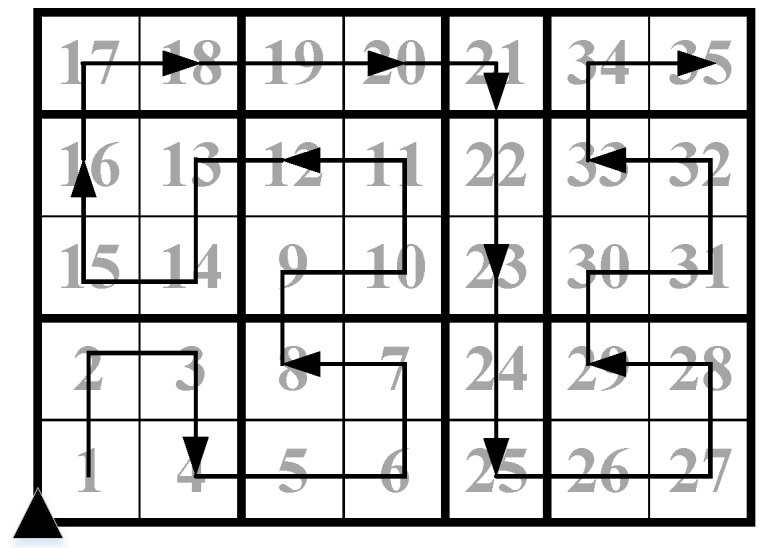
A CH chain in a 5×7 surveillance area.

In Figure 8, we show a CH chain that is generated by Algorithm 1 in a 5×7 surveillance area. The number in each cluster is the chain index, which is assigned in the pseudo Hilbert curve traversing step. By sending the compressed sensed data along the CH chain in the reverse order of cluster indexes, we can collect the compressed sensed data of the whole sensor network in an accumulative manner.

### 4.2. The Distributed Cluster Constructing Algorithm

After generating the CH chain, the sink broadcasts the locations and the indexes of all virtual cluster heads to the network. Since there are usually no sensor nodes deployed in the exact locations of virtual cluster heads, the same number of sensor nodes that are closest to the virtual cluster heads should be selected as CHs on behalf of their clusters. In Section 3, we have restricted that the optimal transmission radius $r^{*}$ of each sensor node should satisfy $r^{*}\geq \sqrt{2/\lambda \pi }$. Hence, at least one sensor node can be guaranteed to reside in the circle with radius $r^{*}$ and centred at the virtual cluster head.

In the distributed cluster-constructing algorithm, the sensor nodes that are in the range of $r^{*}$ from any virtual cluster head will challenge to be CHs. These candidate sensor nodes encapsulate their identifiers and locations into the CH election messages and then broadcast the messages to all sensor nodes within two hops. After a time-out, the sensor nodes that are nearest to the virtual cluster heads will be elected as CHs. Then, these new elected cluster heads broadcast the cluster invitation messages to all sensor nodes within $(D^{*}+1)/2$ hops to construct the intra-cluster data collection structure. After recording or updating the shortest route to a cluster head, each sensor node joins a cluster and points to a parent node in the intra-cluster data collection route. The pseudo code of the distributed cluster-constructing algorithm is summarized in Algorithm 2.

**Algorithm 2** Distributed cluster-constructing algorithm.
**Input:** locations and indexes of virtual CHs {((x,y),i)}, locations of sensor nodes $\left\{\left(\hat{x},\hat{y}\right)\right\}$, $D^{*}$, $r^{*}$**Output:** cluster heads and inter-cluster routes1:The sink broadcasts {((x,y),i)} to all sensor nodes2:**for all**j∈ {sensor nodes} **do**3: **for all** i∈ {virtual CHs} **do**4:  $dist_{ij}=\sqrt{{\left(x_{i}-{\hat{x}}_{i}\right)}^{2}+{\left(y_{i}-{\hat{y}}_{i}\right)}^{2}}$5:  **if** $dist_{ij}\leq r^{*}$ **then**6:   Sensor node *j* broadcasts the CH election message within 2 hops7:  **end if**8:  **if** $dist_{ij}==min\left(dist\left(i,all\;sensor\;nodes\right)\right)$ **then**9:   $CH_{i}=j$10:   Sensor node *j* broadcasts the cluster invitation message within $(D^{*}+1)/2$ hops11:  **end if**12: **end for**13: **if** receives the cluster invitation messages from sensor node *k* **then**14:  **if** hop(CH,k)+1<hop(CH,j) **then**15:   $parent_{j}=k$16:   hop(CH,j)=hop(CH,k)+117:  **end if**18: **end if**19:
**end for**



Note that a new set of cluster heads can be re-elected by following the same rules presented in Algorithm 2 when the structure of the network has changed or new sensor nodes have been added to the network. The residual energy of each sensor node can also be considered as an additional factor to balance the inter-cluster energy consumption in Algorithm 2. Moreover, there is no need to re-generate the CH chain when updating the cluster heads, since the locations and the indexes of the virtual cluster heads are fixed.

## 5. Performance Evaluations

In this section, we evaluate the performance of our proposed collaborative data collection scheme and compare it to the other four data collection schemes through experiments. In the cluster with CS scheme, the whole sensor network is partitioned into a number of clusters based on the number of data transmission, and then, the compressed sensed data are collected along a backbone tree among CHs [37]. In the cluster without CS scheme, the same cluster structure is used, but without compressing the sensed data. In the Shortest Path Tree (SPT) scheme, a shortest path tree is built to collect the plain sensed data in the network. In the SPT with CS scheme, the sensed data are compressed using CS and then transmitted back to the sink along the shortest path tree. We use the total energy consumption, the network lifetime and the distribution of energy consumption as three metrics to compare their performance. Note that the network lifetime is defined as the maximum number of cycles before the first node runs out of energy.

In the experiment, a number of sensor nodes are deployed uniformly and independently in a rectangle surveillance area. We set a sink at the lower-left corner of the surveillance area to collect the sensed data. The number of sensor nodes *n* varies from 200 to 1000. The surveillance area *S* varies from 200 to 600 square meters. The data compression ratio ρ=n/m varies from five to 20. We assume each sensor node is equipped with one 1.5-V 1000-mAh non-rechargeable battery. Therefore, the theoretical initial energy of each sensor node is 5400 J. The energy consumptions of the transmission circuit $E_{elec}$ and the power amplifying circuit $E_{amp}$ to process one bit of data are 50 nJ/bit and 10 pJ/bit/m${}^{2}$, respectively. Furthermore, the TOS_MSG structure in TinyOS is utilized to encapsulate the sensed data transmitted in the network. Therefore, an extra seven bytes of data should be transmitted besides the sensed data.

### 5.1. Performance Analysis

**Figure 9:**
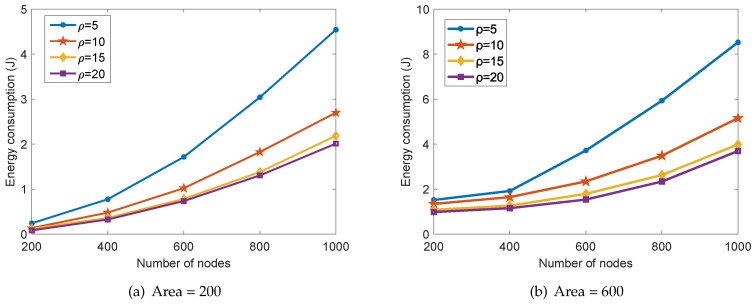
The energy consumption of the proposed scheme with different *n* and ρ.

In the proposed collaborative data collection scheme, the energy consumption in one round of data collection with different number of nodes *n* and data compression ratio ρ is shown in Figure 9. We can see that the total consumed energy increases with the number of nodes *n* and decreases with the compression ratio ρ. This is obvious since a smaller number of sensor nodes and a higher data compression ratio would reduce the number of transmitted sensed data in the network. Furthermore, we can also see that the effect of increasing the compression ratio ρ on the total energy consumption degrades gradually. It should be noted that a higher data compression ratio would also degrade the data reconstruction accuracy according to the compressed sensing theory. By comparing Figure 9a with Figure 9b, we can also conclude that more energy is consumed in a larger surveillance area under the same number of nodes and the compression ratio.

**Figure 10:**
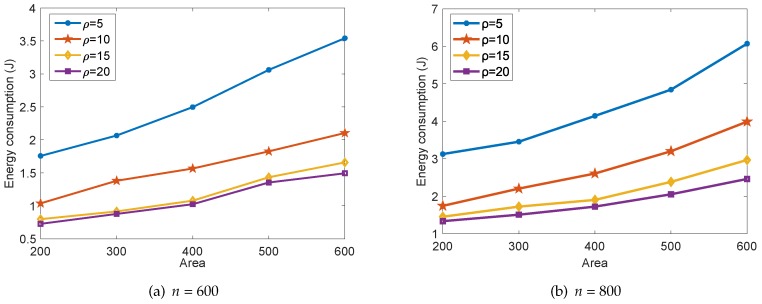
The energy consumption of the proposed scheme with different *S* and ρ.

In Figure 10, we show the energy consumption of our proposed collaborative data collection scheme in one round of data collection with different surveillance area size *S* and compression ratio ρ. Obviously, the expansion of the surveillance area would lead to the increasing of distances between any pair of sensor nodes. Then, the transmitting energy consumption $E_{t}$ of each sensor node in Equation (7) would also increase correspondingly. Furthermore, the total energy consumption decreases with the increase of the data compression ratio ρ, and the effect degrades gradually, which is in accordance with the conclusions drawn from Figure 9. By comparing Figure 10a to Figure 10b, we can also conclude that more energy is consumed when the number of sensor nodes *n* increases. This is in accordance with the conclusions drawn from Figure 9.

### 5.2. Performance Comparison

We evaluate the total energy consumption in one round of data collection of our proposed collaborative data collection scheme and the other four schemes with different numbers of sensor nodes. The consumed energy of the aforementioned five data collection schemes is shown in Table 2. It is obvious that the proposed collaborative data collection scheme is superior to the other four schemes in terms of energy consumption. Moreover, we can also conclude that the larger the number of sensor nodes, the more energy consumed in all data collection schemes. This is due to the fact that more sensed data will be compressed (for our proposed scheme, the cluster with CS scheme and the SPT with CS scheme) and then transmitted back to the sink.

**Figure 11:**
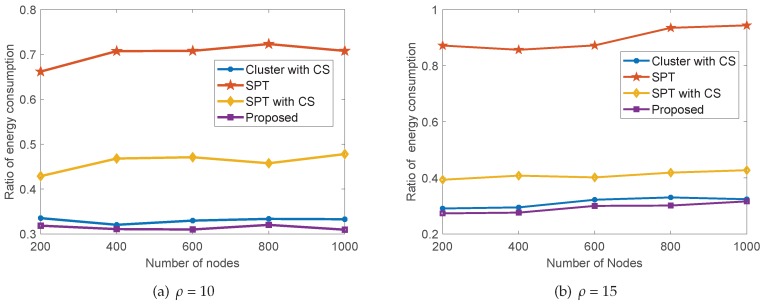
The comparison of the energy efficiency for five schemes.

To further demonstrate the energy efficiency of our proposed collaborative data collection scheme, we take the cluster without CS scheme as the benchmark and evaluate the ratio of the energy consumption of all other schemes compared to that of the benchmark. Note that the cluster without CS scheme is the most energy-intensive scheme, which can be seen clearly from Table 2. We show the comparison of energy efficiency for five schemes in Figure 11, where the compression ratio ρ equals 10 and 15, respectively. We can see that our proposed collaborative data collection scheme presents the highest energy efficiency, followed by the cluster with CS scheme. By comparing the SPT with CS scheme to the SPT scheme and the cluster with CS scheme to the cluster without CS scheme, we can conclude that the compressed sensing theory saves a great deal of energy in the data collection of wireless sensor networks. It is obvious that the top three energy-efficient data collection schemes in our experiment are all CS-based data collection schemes.

**Figure 12:**
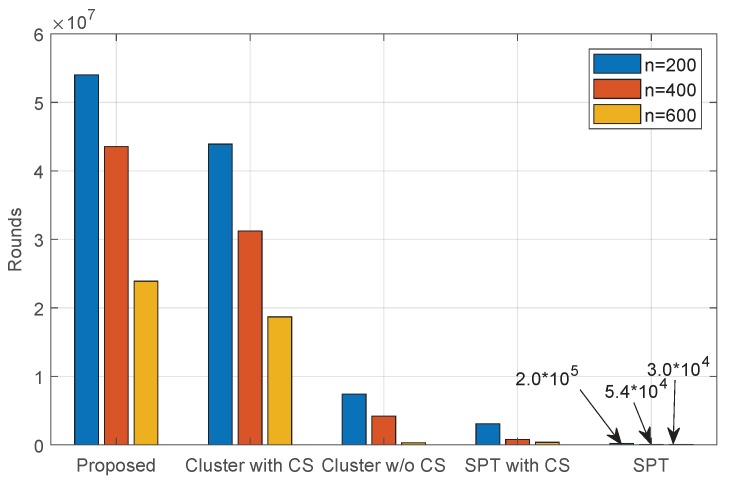
Network lifetime.

However, higher energy efficiency does not mean longer network lifetime directly. Therefore, we record the number of data collection cycles before the first sensor node runs out of energy for all five data collection schemes and report the result in Figure 12. We can see that our proposed collaborative data collection scheme achieves the longest network lifetime among all five data collection schemes. Moreover, the network lifetime of the cluster without CS scheme is longer than that of the SPT scheme, although the latter consumes less energy in one round of data collection. This is due to the unbalanced energy consumption in sensor nodes. Obviously, the sensor nodes that are located closer to the sink need to consume extra energy to forward the sensed data of their children in the data collection tree.

**Figure 13:**
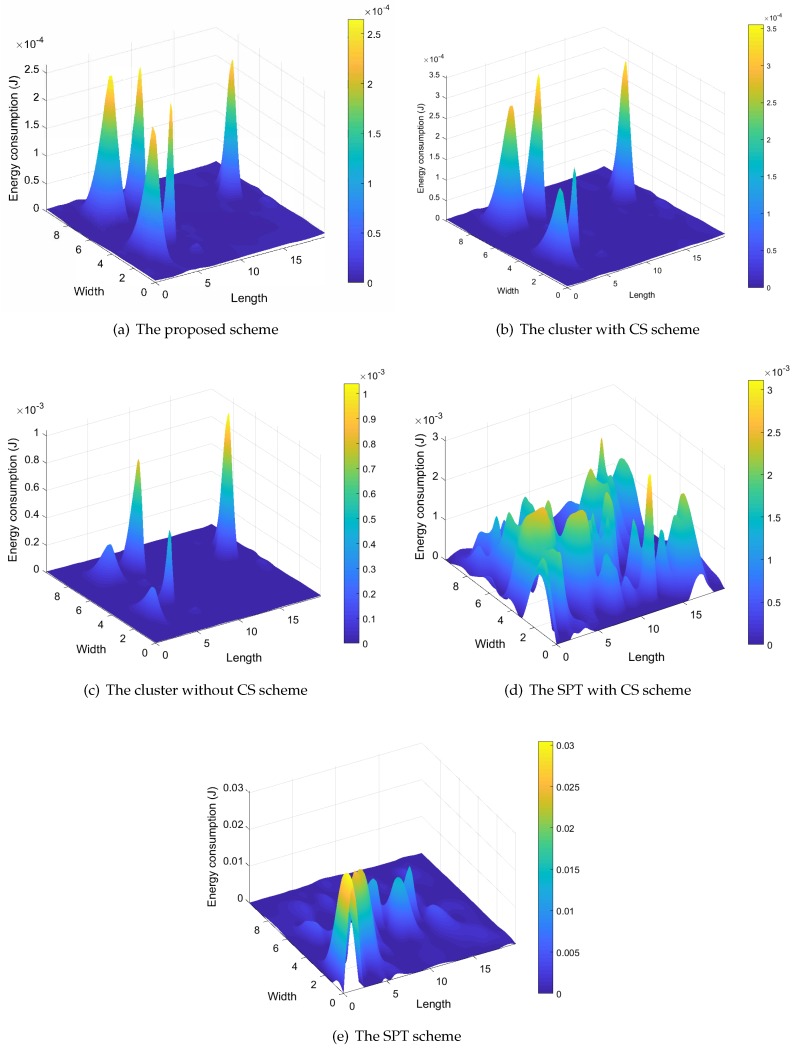
The distribution of the energy consumption.

In order to investigate the distribution of energy consumption in WSNs, we record the consumed energy of all sensor nodes in five data collection schemes and show the result in Figure 13. Firstly, we can see that the distribution of energy consumption in the cluster heads of our proposed scheme is well balanced in comparison to other cluster-based data collection schemes (i.e., the cluster with CS scheme and the cluster without CS scheme). This is due to the fact that the CH chain can provide more balanced energy consumption among cluster heads than the tree-based data collection structure. If we update the cluster heads in all clusters periodically, we can further average the energy consumption of all sensor nodes. Secondly, we can see that the energy consumptions of our proposed scheme, and that of the cluster with CS scheme is obviously lower than those of the other schemes. Therefore, the optimal clustering analysis can provide energy-efficient data collection for WSNs. Finally, the total energy consumptions of non-cluster-based data collection schemes are higher than those of cluster-based schemes, which verifies the advantages of the cluster-based data collection structure.

## 6. Conclusions and Future Research

In this paper, we presented a collaborative data collection scheme based on optimal clustering for wireless sensor networks. By evaluating the intra-cluster energy consumption and the inter-cluster energy consumption individually, we abstracted the optimal clustering problem as a separable convex optimization problem and then solved it to obtain the optimal network parameters. A cluster heads-linking algorithm based on the pseudo Hilbert curve was designed to realize the accumulative inter-cluster data collection. Furthermore, a distributed cluster-constructing algorithm was also designed to construct the intra-cluster data collection structure. Experimental results showed that the proposed scheme outperformed some existing data collection schemes in terms of energy consumption, network lifetime and load balance. In the future, we will plan to extend the proposed scheme with historical data to improve the overall performance. In addition, we will also plan to modify our scheme to monitor the irregular surveillance area.

## Figures and Tables

**Table 1:** The orientation transformation rule.

Parent Orientation	$O_{1}$	$O_{2}$	$O_{3}$	$O_{4}$
I	II	IV	I	I
II	I	II	III	II
III	III	III	II	IV
IV	IV	I	IV	III

**Table 2:** The energy consumption of five schemes in one round of data collection (J). SPT, Shortest Path Tree.

Scheme	Number of Nodes				
	200	400	600	800	1000
Proposed	0.1012	0.3536	0.7777	1.43234	2.0637
Cluster with CS	0.1065	0.3557	0.8078	1.4724	2.1138
Cluster w/oCS	0.3663	1.2067	2.5087	4.4584	6.5277
SPT with CS	0.1441	0.4922	1.2334	2.0445	3.4418
SPT	0.3571	1.0301	2.3671	3.8858	5.6929

## References

[1] Rawat P., Singh K.D., Chaouchi H., Bonnin J.M. (2014). Wireless sensor networks: A survey on recent developments and potential synergies. J. Supercomput..

[2] Borges L.M., Velez F.J., Lebres A.S. (2014). Survey on the characterization and classification of wireless sensor network applications. IEEE Commun. Surv. Tutor..

[3] Yong W., Yang Z., Zhang J., Feng L., Wen H., Shen Y. (2016). CS2-Collector: A new approach for data collection in wireless sensor networks based on two-dimensional compressive sensing. Sensors.

[4] Abdul-Salaam G., Abdullah A.H., Anisi M.H., Gani A., Alelaiwi A. (2016). A comparative analysis of energy conservation approaches in hybrid wireless sensor networks data collection protocols. Telecommun. Syst..

[5] Middya R., Chakravarty N., Naskar M.K. (2016). Compressive sensing in wireless sensor networks—A survey. IETE Tech. Rev..

[6] Luo J., Xiang L., Rosenberg C. Does compressed sensing improve the throughput of wireless sensor networks?. Proceedings of the IEEE International Conference on Communications.

[7] Sucasas V., Radwan A., Marques H., Rodriguez J., Vahid S., Tafazolli R. (2016). A survey on clustering techniques for cooperative wireless networks. Ad Hoc Netw..

[8] Jan B., Farman H., Javed H., Montrucchio B., Khan M., Ali S. (2017). Energy efficient hierarchical clustering approaches in wireless sensor networks: A survey. Wirel. Commun. Mobile Comput..

[9] Singh V.K., Kumar M. (2018). A compressed sensing approach to resolve the energy hole problem in large scale WSNs. Wirel. Pers. Commun..

[10] Singh V.K., Kumar M. (2018). In-network data processing in wireless sensor networks using compressed sensing. Int. J. Sens. Netw..

[11] Lan K.C., Wei M.Z. (2017). A compressibility-based clustering algorithm for hierarchical compressive data gathering. IEEE Sens. J..

[12] Qiao J., Zhang X. (2018). Compressive data gathering based on even clustering for wireless sensor networks. IEEE Access.

[13] Zhao C., Zhang W., Yang Y., Yao S. (2015). Treelet-based clustered compressive data aggregation for wireless sensor networks. IEEE Trans. Veh. Technol..

[14] Li X., Tao X., Mao G. (2017). Unbalanced expander based compressive data gathering in clustered wireless sensor networks. IEEE Access.

[15] Bajwa W., Haupt J., Sayeed A., Nowak R. (2007). Joint source-channel communication for distributed estimation in sensor networks. IEEE Trans. Inf. Theory.

[16] Luo C., Wu F., Sun J., Chen C.W. Compressive data gathering for large-scale wireless sensor networks. Proceedings of the International Conference on Mobile Computing and Networking.

[17] Xiang L., Luo J., Rosenberg C. (2013). Compressed data aggregation: energy-efficient and high-fidelity data collection. IEEE/ACM Trans. Netw..

[18] Zheng H., Yang F., Tian X., Gan X., Wang X., Xiao S. (2015). Data gathering with compressive sensing in wireless sensor networks: A random walk based approach. IEEE Trans. Parallel Distrib. Syst..

[19] Hammoudeh M., Newman R. (2015). Information extraction from sensor networks using the Watershed transform algorithm. Inf. Fusion.

[20] Quan L., Xiao S., Xue X., Lu C. (2016). Neighbor-aided spatial-temporal compressive data gathering in wireless sensor networks. IEEE Commun. Lett..

[21] Cheng J., Ye Q., Jiang H., Wang D. (2013). STCDG: An efficient data gathering algorithm based on matrix completion for wireless sensor networks. IEEE Trans. Wirel. Commun..

[22] Piao X., Hu Y., Sun Y., Yin B., Gao J. (2014). Correlated spatio-temporal data collection in wireless sensor networks based on low rank matrix approximation and optimized node sampling. Sensors.

[23] Xie K., Ning X., Wang X., Xie D., Cao J., Xie G., Wen J. (2017). Recover corrupted data in sensor networks: A matrix completion solution. IEEE Trans. Mobile Comput..

[24] Candes E.J., Wakin M.B. (2008). An introduction to compressive sampling. IEEE Signal Process. Mag..

[25] Donoho D.L. (2006). Compressed sensing. IEEE Trans. Inf. Theory.

[26] Qaisar S., Bilal R.M., Iqbal W., Naureen M. (2013). Compressive sensing: From theory to applications, a survey. J. Commun. Netw..

[27] Campobello G., Segreto A., Serrano S. (2016). Data gathering techniques for wireless sensor networks: A comparison. Int. J. Distrib. Sens. Netw..

[28] Candes E., Romberg J., Tao T. (2006). Robust uncertainty principles: Exact signal reconstruction from highly incomplete frequency information. IEEE Trans. Inf. Theory.

[29] Figueiredo M.A.T., Nowak R.D., Wright S.J. (2008). Gradient projection for sparse reconstruction: Application to compressed sensing and other inverse problems. IEEE J. Sel. Top. Signal Process..

[30] Soussen C., Idier J., Duan J., Brie D. (2014). Homotopy based algorithms for l0-regularized least-squares. IEEE Trans. Signal Process..

[31] Jain P., Tewari A., Dhillon I.S. (2017). Partial hard thresholding. IEEE Trans. Inf. Theory.

[32] Han G., Xu H., Duong T.Q., Jiang J., Hara T. (2013). Localization algorithms of wireless sensor networks: A survey. Telecommun. Syst..

[33] Xu G., Zhu M., Luo X., Wu M., Ren F. (2012). An unequal clustering algorithm based on energy balance for wireless sensor networks. IEEJ Trans. Electr. Electron. Eng..

[34] Li H., Liu Y., Chen W., Jia W., Li B., Xiong J. (2013). COCA: Constructing optimal clustering architecture to maximize sensor network lifetime. Comput. Commun..

[35] Zhang J., Feng X., Liu Z. (2018). A grid-based clustering algorithm via load analysis for industrial Internet of things. IEEE Access.

[36] Wu C., Chang Y. (2012). Approximately even partition algorithm for coding the Hilbert curve of arbitrary-sized image. IET Image Process..

[37] Xie R., Jia X. (2014). Transmission-efficient clustering method for wireless sensor networks using compressive sensing. IEEE Trans. Parallel Distrib. Syst..

